# Mandibular full-arch fixed prostheses supported by three-dental-implants: A protocol of an overview of reviews

**DOI:** 10.1371/journal.pone.0265491

**Published:** 2022-04-04

**Authors:** Kelvin I. Afrashtehfar, Rosalin A. Moawad, Afaf W. F.-Eddin, Hom-Lay Wang

**Affiliations:** 1 Evidence-Based Practice Unit, Clinical Sciences Department, College of Dentistry, Ajman University, Ajman City, Ajman Emirate, UAE; 2 Department of Reconstructive Dentistry and Gerodontology, School of Dental Medicine, Faculty of Medicine, University of Bern, Bern, Switzerland; 3 Centre of Medical and Bio-allied Health Sciences Research (CMBHSR), Ajman University, Dubai, City of Gold, UAE; 4 Department of Periodontics and Oral Medicine, School of Dentistry, University of Michigan, Ann Arbor, Michigan, United States of America; Universidade Federal Fluminense, BRAZIL

## Abstract

**Introduction:**

To minimize trauma and cost of treatment, oral health practitioners have successfully rehabilitated full arches by supporting the prostheses on four implants. However, there is no consensus whether less than four implants supporting full mandibular arches would provide similar clinical outcomes to other well-established all-on-four alternative.

**Objective:**

To identify, summarize, appraise, and compare the clinical outcomes evidence of three-implant fixed full-arch prostheses in completely edentulous mandibular patients.

**Materials and methods:**

This overview of systematic reviews (OoSRs) will include secondary synthesis studies (i.e., systematic reviews with or without a meta-analysis). A three-step search strategy will be conducted in MEDLINE (Ovid), EMBASE (Ovid), Cochrane Database of Systematic Reviews, Scopus, Web of Science (WoS Core Collection), and Google Scholar. Grey literature and a manual search in 12 specialized journals will also be conducted. Three independent reviewers will screen all retrieved articles for eligibility, extract data and assess the methodological quality of the included studies. The results will be presented as tables or narrative synthesis. The studies will be evaluated for risk of bias by ROBIS and methodology quality by the AMSTAR-2 tool. If new primary studies are identified, a meta-analysis will be conducted. Certainty of the evidence will be assessed to answer the following focused research question: In edentulous mandibular patients, what are the implant and prostheses clinical outcomes of three-implant fixed full-arch prostheses compared to other all-on-x solutions?

**Discussion:**

There are some systematic reviews about the use of fixed complete dental prostheses supported by three implants; however, their clinical outcomes related to the other all-on-four plus solutions are conflicting. So, an overview on this topic is required to provide recommendations.

**Review registration number:**

International prospective register of systematic reviews (PROSPERO) ID#: CRD42021262175. National Institute for Health Research (NIHR) and Centre for Reviews and Dissemination, University of York, York, United Kingdom.

## Introduction

Edentulism is a global phenomenon that is described as the complete loss of all teeth [1–3]. Edentulism is a common disability within the aging population since edentulism increases with age [4, 5]. An increase overall in the aging population has increased the number of edentulous Americans [6]. It is estimated that the number of edentulous Brazilians is expected by 2040 to increase in their aging population to > 64 million [7]. Consequently, it is globally projected that the need to reconstruct edentulous patients will intensify the future [3].

There is certain grade of alveolar bone atrophy in most edentulous aging population [8]. The mandibular anterior region crucial structures are the extension of the anterior loop, mandibular canal/inferior alveolar nerve, and mental nerve [9]. The mandibular posterior region may present four branching patterns of the inferior alveolar canal (IAC) branches [10]. Consequently, familiarity of the patient’s anatomical structures and variations permit the dental clinician propose an adequate treatment plan [10–12].

Mandibular fixed and removable full-arch implant prostheses present high implant and prosthesis survival rates, even when immediate loaded [13–15]. Indeed, immediate loaded implants may present long-term success rate [16]. Regarding the removable options, a recent consensus statement that provided clinical recommendations for implant-overdentures (IODs) determined 4 implants have been effectively used for mandibular IODs and 2 implants are considered the minimum standard of care treatment [13]. Mandibular implant fixed full-arch prostheses produce high quality of life (QoL) and patient satisfaction concerning retention, stability, and ease of chewing [13, 14, 17]. Interestingly, there are no differences in overall QoL or patient satisfaction between mandibular fixed and removable full-arch implant prostheses [13, 14]. A recent meta-analysis advocate that full-arch prostheses supported by short implants in atrophic edentulous mandibles may be a feasible treatment alternative [18]. However, long-term data is needed to support this recommendation. People mostly prefer a mandibular 4-implant-supported fixed full-arch prostheses when compared to conventional complete dentures or 2-implant-retained overdentures (IOD) [19]. Interestingly, people with less school education and those with lower financial income are less inclined to select implant-supported fixed full-arch prostheses [19].

There is often controversy regarding the number if implants needed for mandibular implant fixed full-arch prostheses. However, the number of implants and the distribution are determined by the type of prosthesis. Moreover, the type of prosthesis is heavily influenced by the patients’ finances [13]. Regarding the current literature for mandibular implant fixed full-arch prostheses, there is some evidence for indicating more than 6 implants whereas there is a strong evidence of high survival implant and prosthesis rates for five and four implants [13]. A meta-analysis found similar survival rates of mandibular implant fixed full-arch prostheses supported by < 5 implants when compared to > 4 implants [20]. Fixed full-arch prostheses supported by 4 implants or the all-on-four concept comprises increasing the prosthesis length by tilting the distal implants [21]. Consequently, fewer number of implants are needed in the posterior region, which also compensates for severe bone resorption The same is true for an all-on-three approach introduced in 1999 as the Novum protocol by Brånemark et al. [22]. They reported a mean implant and prothesis survival of 98% after a 3-year follow-up in 50 patients. The reported benefits of an all-on-three approach are lower cost, fewer clinical appointments, and reduced total treatment time [22–26]. Interestingly, a meta-analysis [27] explored the clinical outcomes of fixed full-arch prostheses supported by less than five implants. However, this specific study did not include sufficient primary studies reporting 3-implant solutions data [27]. Therefore, the controversies remain on if three implants are adequate to support fixed full-arch prostheses [28, 29].

In view of the expected growth of fully edentulous arch patients, awareness of the fixed treatment options supported with fewer number of implants than traditionally accepted could contribute to permitting potential patients to have access to full-arch fixed implant prostheses, especially in low- and medium-income population in need. In this manner, a better knowledge of the medium- and long-term survival of mandibular fixed full-arch prostheses supported by a minimum number of dental implants may be helpful in the adoption of interventions to reduce the financial investment burden, and in the reduction of the perpetuated disparities in the access to fixed full-arch prostheses between privileged and underprivileged groups.

Secondary studies have assessed the implant and prosthetic survival of mandibular fixed full-arch prostheses supported by three implants in adult patients compared to similar interventions supported by more than three implants, but the results of such studies seem inconclusive. A preliminary search of PROSPERO, MEDLINE (Ovid), MEDLINE (PubMed), EMBASE (Ovid), Cochrane Database of Systematic Reviews, was conducted, and no overview of systematic reviews (OoSRs) on this subject was found. Therefore, the objective of this OoSRs is to identify, summarize, appraise, and compare the clinical outcomes evidence of three-implant fixed full-arch prostheses in completely edentulous mandibular patients.

### Focused review question

In edentulous mandibular patients, what are the implant and prosthetic outcomes of three-implant-supported fixed full-arch prostheses compared to other all-on-4 and plus solutions?

## Materials and methods

The proposed OoSRs will be conducted in accordance with Preferred Reporting Items for OoSRs (PRIO-harms) to promote a more balanced reporting of benefits and harms in OoSRs of health care interventions [30]. OsSRs facilitate adequate decision making by comparing systematic reviews and/or meta-analyses and promote a critical analysis of the main viable evidence about a particular issue [31–34]. The Preferred Reporting Items for Systematic review and Meta-Analysis Protocols (PRISMA-P) checklist [35, 36] was applied for the development of this protocol (S1 Table).

### Overview registration number

The protocol has been registered in International prospective register of systematic reviews (PROSPERO: CRD42021262175). A submission identification number (262175) was provided by the National Institute for Health Research (NIHR) on June 20th, 2021.

### Inclusion criteria

#### Participants

The participants or populations being studied by the OoSRs include adults with a fixed dental reconstruction supported by three implants to replace the missing teeth of their edentulous mandible. The participants included in the SRs should be properly described concerning characteristics such as age, sex, sample size, follow-up (mean, standard deviation, range), and clinical conditions (implant [bone quality, bone quantity/volume, medication/drugs, peri-implant bone loss, suppuration, bleeding in probing, peri-implany mucositis absence/presence, peri-implantitis absence/presence] and prosthetic characteristics [screwed/cemented retention, cantilever length, prosthetic material]). Secondary studies investigating implant-supported fixed reconstructions other than complete dental prostheses (i.e., implant-supported fixed partial dentures) will be excluded.

#### Intervention

In this OoSRs, the nature of the interventions or the exposures to be reviewed are defined as fixed full-arch prostheses supported by three dental implants. SRs will be included if they are detailed in the study design: baseline and implant and prosthetic follow-ups, whether the groups were conducted in parallel or cross-over, and if there were associations with other factors that could interfere with the survival of the treatment (e.g., systemic conditions, low bone quality/quantity, drugs).

#### Control

The selected SRs should demonstrate that the interventions were compared with a control group composed of anther all-on-4 and plus solutions (e.g., participants who had their edentulous mandibles restored with complete fixed dental prostheses supported by 4, 5, 6, 7, 8 or more osseointegrated implants).

#### Outcome

This OoSRs will consider as prosthetic and implant survival from baseline to the last available follow-up as main outcomes. Prosthetic and implant success, and complications or adverse events, as well as crestal bone loss and patient satisfaction, will be considered as secondary outcomes. Additionally, secondary predictors of peri-implant disease outcomes (e.g., bleeding index and plaque index) will be reported.

The metric system will be the measurement unit for reporting the data of each of the outcomes of interest of this overview. Conversion will be performed if the included secondary studies have used a different measurement unit (i.e., Imperial system).

#### Types of studies

SRs and MAs of observational Studies (OSs) and randomized controlled trials (RCTs) will be included. OoSRs, scoping reviews, rapid reviews, critical reviews, mapping reviews, mixed-methods reviews, systematized reviews, systematic search and reviews, state-of-the-art reviews, narrative reviews, letters to the editor, editorials, case reports, and case series will be excluded.

#### Clinical settings

No restriction will be placed regarding the clinical setting (e.g., private practice or public offices). Studies in low-, middle- and high-income countries will be considered.

### Search strategy

For the creation of the search strategy for this OoSRs, three steps were taken. First, a preliminary search was implemented in MEDLINE (PubMed) to detect records that meet the inclusion criteria. Subsequently, the titles and abstracts of these records were used to pinpoint keywords and indexing terms to finalize the definitive search strategy. Secondly, the final search strategy was tailored by KIA for MEDLINE (Ovid) using MeSH terms, entry terms, and synonyms linked with Boolean operators “AND” and “OR” (*see* S1 File). The search was adjusted for each of the other databases, using controlled vocabulary (MeSH, EMTREE, and others), as well as entry terms. Lastly, the list of references of all records included in the SRs and MAs will be screened. Before final analysis, the searches will be updated. No restrictions are planned to be applied to language or year of publication, and geographic limits.

A manual search for relevant publications in 12 specialized journals, *Clinical Oral Implants Research*, *Clinical Implant Dentistry and Related Research*, *Japanese Dental Science Review*, *Journal of Prosthodontic Research*, *Clinical Oral Investigations*, *Journal of Prosthetic Dentistry*, *Journal of Evidence-Based Dental Practice*, *The International Journal of Oral & Maxillofacial Implants*, *Journal of Prosthodontics*, *Implant Dentistry*, *Journal of Applied Oral Science*, and *Journal of Periodontal & Implant Science* from 2010 up to 2021 will also be performed.

### Information sources

Searches will be conducted in the following electronic databases: MEDLINE (via Ovid), EMBASE (via Ovid), Cochrane Database of Systematic Reviews (via Cochrane Library), Scopus, and Web of Science (via WoS Core Collection). Grey literature will be searched in OpenGrey (opengrey.eu), Networked Digital Library of Theses and Dissertations (ndltd.org), and ProQuest Dissertations & Theses Global (via ProQuest). Additionally, a search in Google Scholar limited to the first 100 most relevant studies [37] will also be conducted.

### Study selection

All identified citations will be uploaded into EndNote Web (version X9, Clarivate Analytics, PA, USA) and duplicates eliminated. Three reviewers will select the records independently. The reviewers will begin by screening the titles/abstracts. The records that meet the inclusion criteria after consensus will be selected. If the title/abstract does not provide enough data for a decision, the full text of the record will be evaluated. Records whose full text fulfills the eligibility criteria will also be considered. Any disagreements arising during the study selection will be discussed and resolved by consensus. Interrater reliability will be neglected as this does add any benefit to the present OoSRs-harms/interventions. If the OsSRs was focused on a diagnostic outcome, an estimation of kappa Cohen would have been planned. Nevertheless, the levels of agreement will be conducted with 95%CI in the selection process of the secondary studies included.

For a better visualization of each stage of the study selection, a flow diagram will be generated as indicated by the PRISMA (Preferential Reporting Items for Systematic Reviews and Meta-analyses) guidelines [38–41].

### Data extraction

Data extraction will be conducted independently by two reviewers (RAW, AWF), and this will be revised by one reviewer (KIA). If disagreements between reviewers arise, a discussion until consensus is reached will be performed. Data extracted will include study details (author, year, journal, number of primary studies included, funding, language, and databases searched), study methods (design, setting, sample, recruitment, intervention, comparators), dropouts (reason for dropouts and dropout rate), assessment of the implant and prosthetic outcomes (survival rate, success rate) the patient-reported outcome measures (PROMs), and main adverse events. The form developed for data extraction is displayed as supporting information (S2 File). The primary studies analyzed in SRs will be consulted for completion if some important data is missing.

### Assessment of methodological quality

Three reviewers will assess the risk of bias and methodological quality of the included records independently [42, 43]. The risk of bias will be appraised using the ROBIS tool, according to specific guidelines for SRs related to interventions [44]. The methodological quality of the SRs will be assessed using the AMSTAR 2 tool (https://amstar.ca/docs/AMSTAR-2.pdf). Differences occurring during will be discussed until a resolution is reached by consensus.

All studies will be submitted to data extraction and synthesis. The influence of risk of bias and methodological quality will be considered when generating conclusions and recommendations. The levels of agreement will be performed with 95%CI in both quality assessment tools used of the secondary studies included.

#### The ROBIS tool

The ROBIS tool consists of three phases [44, 45]. Phase 1 is the assessment of relevance, which is considered optional. The phase 2 is the identification of bias during the review conduction, which is performed by four domains: study eligibility criteria, identification and selection of studies, data collection and study appraisal, and synthesis and findings. Phase 3 is the judgment of the risk of bias through the summary of phase 2 findings. All phases except phase 1, have five options to answer each sub-area of the domains that vary in (Y) “yes,” (PY) “probably yes,” (PN) “probably not,” (N) “no,” and (NI) “not informed.” The reason for concern is the risk of bias classified as high, low, or unclear.

#### The AMSTAR-2 tool

The AMSTAR 2 tool consists of 16 domains where seven are considered critical for the reliability of SRs findings (i.e., domains 2, 4, 7, 9, 11, 13, and 15), ranking the SR in high, moderate, low, and critically low [46, 47]. For each domain, the answers will vary in “yes” (the domain criteria have been fulfilled), “partially yes” (the domain criteria have been partially fulfilled), and “no” (the domain criteria have not been fulfilled). Several weaknesses (i.e., more than 3) in non-critical domains will reduce confidence in results. Thus, in this case, the classification starts from moderate instead of high level.

### Data synthesis

In the first step of data synthesis, the results of the studies selection process will be displayed using the PRISMA flowchart [40, 41]. In the second step of data synthesis, the findings of the SRs and MAs will be summarized, and the characteristics of the variables will be described in tables or in narrative form where each one will be classified and, if suitable, allocated as a statistical measure. This will facilitate the of studies comparison, effects visualization and findings critical interpretation aided by the RevMan 5.4 (Copenhagen, The Nordic Cochrane Centre, Cochrane) [48]. The overlap of studies among the SRs included will be estimated using the calculation of the corrected covered area (CCA) [48]. If there is a high overlap of studies among the SRs, the most recent SRs will be kept and the impact of such findings on the evidence will be discussed.

A search for primary studies will be conducted at the end of this project and if new relevant trials are identified, a meta-analysis will be conducted. In this case, effect measures will be displayed as mean differences (for continuous data) or odds ratio/risk ratio (for dichotomous data) and the 95% confidence interval (CI) will be calculated. Retrospective and prospective primary studies will separately be analyzed for each method [49]. Heterogeneity will be reported by applying the standard x^2^ and *I*^2^ tests. If heterogeneity is equal to or greater than 40%, a random effect model will be chosen, whereas if heterogeneity is less than 40%, a fixed-effect model would be selected. To reduce heterogeneity, sensitivity analysis would be conducted removing studies one by one. Subgroup analyses will be conducted if possible. The included studies design will also be considered during the subgroup’s analysis. Forest plots will visually aid presenting the meta-analysis findings and a funnel plot to presenting the publication bias [50–52].

## Supporting information

S1 Table PRISMA-P 2015 checklist: Recommended items to address in a systematic review protocol. (PDF)

S1 File Search conducted in Medline (Ovid) on June 18th, 2021. (PDF)

S2 File Data extraction form of included secondary studies. (PDF)
