# Supplementary material for: Mandibular full-arch fixed prostheses supported by three-dental-implants: A protocol of an overview of reviews
Source: PLoS One. 2022 Apr 4;17(4):e0265491. doi: 10.1371/journal.pone.0265491 (PMC8979460; doi:10.1371/journal.pone.0265491)
Supplement: S1 Table — (PDF) [file pone.0265491.s001.pdf]

S1 Table. PRISMA-P 2015 checklist: recommended items to address in a systematic review protocol*

Section and topic	Item No	Checklist item	Manuscript location
**ADMINISTRATIVE INFORMATION**			
Title:			
Identification	1a	Identify the report as a protocol of a systematic review	Line 2
Update	1b	If the protocol is for an update of a previous systematic review, identify as such	NA
Registration	2	If registered, provide the name of the registry (such as PROSPERO) and registration number	Line 128
Authors:			
Contact	3a	Provide name, institutional affiliation, e-mail address of all protocol authors; provide physical mailing address of corresponding author	Lines 5-15
Contributions	3b	Describe contributions of protocol authors and identify the guarantor of the review	Lines 14-18
Amendments	4	If the protocol represents an amendment of a previously completed or published protocol, identify as such and list changes; otherwise, state plan for documenting important protocol amendments	NA
Support:			
Sources	5a	Indicate sources of financial or other support for the review	Lines 271-274
Sponsor	5b	Provide name for the review funder and/or sponsor	Lines 272-273
Role of sponsor or funder	5c	Describe roles of funder(s), sponsor(s), and/or institution(s), if any, in developing the protocol	Lines 273-274
**INTRODUCTION**			
Rationale	6	Describe the rationale for the review in the context of what is already known	Lines 10-112
Objectives	7	Provide an explicit statement of the question(s) the review will address with reference to participants, interventions, comparators, and outcomes (PICO)	Lines 112-114, 130-133
**METHODS**			
Eligibility criteria	8	Specify the study characteristics (such as PICO, study design, setting, time frame) and report characteristics (such as years considered, language, publication status) to be used as criteria for eligibility for the review	Lines 145-169
Information sources	9	Describe all intended information sources (such as electronic databases, contact with study authors, trial registers or other grey literature sources) with planned dates of coverage	Lines 180-195
Search strategy	10	Present draft of search strategy to be used for at least one electronic database, including planned limits, such that it could be repeated	Lines 172-180, S1 File

Study records:			
Data management	11a	Describe the mechanism(s) that will be used to manage records and data throughout the review	Lines 197-204
Selection process	11b	State the process that will be used for selecting studies (such as two independent reviewers) through each phase of the review (that is, screening, eligibility and inclusion in meta-analysis)	Lines 199-201, 259-262
Data collection process	11c	Describe planned method of extracting data from reports (such as piloting forms, done independently, in duplicate), any processes for obtaining and confirming data from investigators	Lines 211-221, S2 File
Data items	12	List and define all variables for which data will be sought (such as PICO items, funding sources), any pre-planned data assumptions and simplifications	Lines 136-160, 205-206
Outcomes and prioritization	13	List and define all outcomes for which data will be sought, including prioritization of main and additional outcomes, with rationale	Lines 156-160, 217-219
Risk of bias in individual studies	14	Describe anticipated methods for assessing risk of bias of individual studies, including whether this will be done at the outcome or study level, or both; state how this information will be used in data synthesis	Lines 223-247
Data synthesis	15a	Describe criteria under which study data will be quantitatively synthesised	Lines 255-256, 259-260
	15b	If data are appropriate for quantitative synthesis, describe planned summary measures, methods of handling data and methods of combining data from studies, including any planned exploration of consistency (such as I^2, Kendall's τ)	Lines 260-266
	15c	Describe any proposed additional analyses (such as sensitivity or subgroup analyses, meta-regression)	Lines 265-267
	15d	If quantitative synthesis is not appropriate, describe the type of summary planned	Lines 250-258
Meta-bias(es)	16	Specify any planned assessment of meta-bias(es) (such as publication bias across studies, selective reporting within studies)	Lines 266-269
Confidence in cumulative evidence	17	Describe how the strength of the body of evidence will be assessed (such as GRADE)	Lines 245-247

*** It is strongly recommended that this checklist be read in conjunction with the PRISMA-P Explanation and Elaboration (cite when available) for important clarification on the items. Amendments to a review protocol should be tracked and dated.** **The copyright for PRISMA-P (including checklist) is held by the PRISMA-P Group and is distributed under a Creative Commons Attribution Licence 4.0.**

From: Shamseer L, Moher D, Clarke M, Ghersi D, Liberati A, Petticrew M, Shekelle P, Stewart L, PRISMA-P Group. Preferred reporting items for systematic review and meta-analysis protocols (PRISMA-P) 2015: elaboration and explanation. BMJ. 2015 Jan 2;349(jan02 1):g7647.
