# Supplementary material for: Mandibular full-arch fixed prostheses supported by three-dental-implants: A protocol of an overview of reviews
Source: PLoS One. 2022 Apr 4;17(4):e0265491. doi: 10.1371/journal.pone.0265491 (PMC8979460; doi:10.1371/journal.pone.0265491)
Supplement: S1 File — (PDF) [file pone.0265491.s002.pdf]

S1 File. Search conducted in Medline (Ovid) on June 18th, 2021.

Search	Query	Recorded retrieved
#1	exp Dental Implants/ OR Dental implant*.mp. OR exp Dental Implantation/ OR all-on-three.mp. OR Trefoil.mp.	46,024
#2	exp Dental Prosthesis/ OR (dental and prosthes*).mp. [mp=title, abstract, original title, name of substance word, subject heading word, floating sub-heading word, keyword heading word, organism supplementary concept word, protocol supplementary concept word, rare disease supplementary concept word, unique identifier, synonyms]	115,157
#3	exp "systematic review"/ OR exp meta-analysis/ OR systematic review.m_titl. OR meta-analysis.m_titl.	285,567
#4	exp Survival/ OR survival.mp.	1,324,496
#5	((#1) AND (#2) AND (#3) AND (#4))	193
