# Supplementary material for: Mandibular full-arch fixed prostheses supported by three-dental-implants: A protocol of an overview of reviews
Source: PLoS One. 2022 Apr 4;17(4):e0265491. doi: 10.1371/journal.pone.0265491 (PMC8979460; doi:10.1371/journal.pone.0265491)
Supplement: S2 File — (PDF) [file pone.0265491.s003.pdf]

S2 File. Data extraction form of included secondary studies.

Reviewer ID: _______________ Date: __/__/____

Study details
First author:
Year:
Country:
Journal:

Study method/ characteristics
Study aim:
Focused research question:
Number of included studies for qualitative analysis:
Number of included studies for quantitative analysis:
Total number of included randomised studies:
Other included primary studies:
Number of searched databases:
Language restriction:
Date restriction:
Grey literature:
Hand search:
Reference lists searched:
Date of last search:
Date of available online (ahead-of-pub):
Review inclusion criteria:
Review exclusion criteria:

Protocol registration details:
The date of first submission for registration consideration:
The date of protocol registration:
Ethical approval details:

Methodological or risk assessment tool applied to primary studies:
Publication bias details:
Funding source:
Authors' declaration:

Participants
Sample size:
Age (mean and range):
Sex: Male (n; %) _____ Female (n; %) ________
Inclusion criteria:
Exclusion criteria:
Baseline imbalances reported:

Severity of illness reported:
Co-morbidities reported:
Other relevant sociodemographic reported:
Subgroups measured:
    Primary studies countries/location:
    Recruitment procedures reported:

Intervention group

	Statement in the paper	Location in the text or source *(page, figure, table)*
Description		
Follow-up (mean and range)		
Settings		
Co-interventions		
Compliance		
Dental implant characteristics		
Implant placement protocol		
Prothesis loading protocol		
Prothesis retention mechanism		

Dependent variable (outcome)
Primary outcome measures:
Primary outcomes definition:
Secondary outcome measures:
Adverse effects:
Authors' conclusion:

Data analysis
Statistical methods:
Meta-analysis:
Effect measure:

Reviewer comments
